# Screening models using multiple markers for early detection of late-onset preeclampsia in low-risk pregnancy

**DOI:** 10.1186/1471-2393-14-35

**Published:** 2014-01-20

**Authors:** Hee Jin Park, Soo Hyun Kim, Yong Wook Jung, Sung Shin Shim, Ji Yeon Kim, Yeon Kyung Cho, Antonio Farina, Margherita Zanello, Kyoung Jin Lee, Dong Hyun Cha

**Affiliations:** 1 Department of Obstetrics and Gynecology, CHA Gangnam Medical Center, CHA University, Seoul, Republic of Korea; 2 Department of Medicine and Surgery (DIMEC), Division of Prenatal Medicine, University of Bologna, Bologna, Italy; 3 Department of Obstetrics and Gynecology, CHA Gangnam Medical Center, 650-9 Yeoksam-dong, Gangnam-gu, Seoul, Republic of Korea

**Keywords:** Preeclampsia, Soluble fms-like tyrosine kinase, Placental growth factor, sFlt-1, PlGF, sFlt-1/PlGF ratio, PAPP-A, BMI, Low-risk population

## Abstract

**Background:**

Our primary objective was to establish a cutoff value for the soluble fms-like tyrosine kinase 1(sFlt-1)/placental growth factor (PlGF) ratio measured using the Elecsys assay to predict late-onset preeclampsia in low-risk pregnancies. Our secondary objective was to evaluate the ability of combination models using Elecsys data, second trimester uterine artery (UtA) Doppler ultrasonography measurements, and the serum fetoplacental protein levels used for Down’s syndrome screening, to predict preeclampsia.

**Methods:**

This prospective cohort study included 262 pregnant women with a low risk of preeclampsia. Plasma levels of pregnancy-associated plasma protein-A (PAPP-A) and serum levels of alpha-fetoprotein, unconjugated estriol, human chorionic gonadotropin, and inhibin-A were measured, and sFlt-1/PlGF ratios were calculated. All women underwent UtA Doppler ultrasonography at 20 to 24 weeks of gestation.

**Results:**

Eight of the 262 women (3.0%) developed late-onset preeclampsia. Receiver operating characteristic curve analysis showed that the third trimester sFlt-1/PlGF ratio yielded the best detection rate (DR) for preeclampsia at a fixed false-positive rate (FPR) of 10%, followed by the second trimester sFlt-1/PlGF ratio, sFlt-1 level, and PlGF level. Binary logistic regression analysis was used to determine the five best combination models for early detection of late-onset preeclampsia. The combination of the PAPP-A level and the second trimester sFlt-1/PlGF ratio yielded a DR of 87.5% at a fixed FPR of 5%, the combination of second and third trimester sFlt-1/PlGF ratios yielded a DR of 87.5% at a fixed FPR of 10%, the combination of body mass index and the second trimester sFlt-1 level yielded a DR of 87.5% at a fixed FPR of 10%, the combination of the PAPP-A and inhibin-A levels yielded a DR of 50% at a fixed FPR of 10%, and the combination of the PAPP-A level and the third trimester sFlt-1/PlGF ratio yielded a DR of 62.5% at a fixed FPR of 10%.

**Conclusions:**

The combination of the PAPP-A level and the second trimester sFlt-1/PlGF ratio, and the combination of the second trimester sFlt-1 level with body mass index, were better predictors of late-onset preeclampsia than any individual marker.

## Background

Preeclampsia is characterized by hypertension and significant proteinuria during pregnancy. This multisystem disorder occurs in approximately 3% of pregnancies [1], and can progress to eclampsia with life-threatening seizures.

Recent evidence indicates that there are numerous phenotypes of preeclampsia, indicating that a number of pathophysiologic mechanisms related to the mother and fetus may contribute to the timing of disease onset [2]. Preeclampsia has been described as two distinct disease entities: early-onset preeclampsia, which develops before 34 weeks of gestation, and late-onset preeclampsia, which develops at or after 34 weeks of gestation [3]. Early-onset preeclampsia is strongly associated with deficient trophoblast invasion and failure of normal spiral artery remodeling. Late-onset preeclampsia may be caused by increased maternal vascular susceptibility to the normal inflammatory state of pregnancy or atherosis of a placenta that initially developed normally [4].

Preeclampsia is one of the leading causes of maternal mortality worldwide, and in developed countries it increases perinatal mortality five-fold [5]. Chronic hypertension, pregestational diabetes mellitus, multifetal pregnancy, and preeclampsia during a previous pregnancy are associated with a three- to five-fold increased risk of preeclampsia [6]. This high-risk population tends to develop severe or early preeclampsia, and accounts for about one-third of cases of early-onset preeclampsia [7]. Patients at high risk of preeclampsia usually undergo close surveillance during pregnancy, and can be referred to specialized centers for perinatal care. Women at low risk of preeclampsia also need close surveillance, and detection and prompt management of these patients can improve maternal and fetal mortality and morbidity. However, there is no reliable screening test for early identification of late-onset preeclampsia in low-risk pregnancies. Combination of the clinical, biochemical, and biophysical markers is likely to increase the predictive power of screening examinations.

It has been suggested that placental soluble fms-like tyrosine kinase 1 (sFlt-1) is a causative factor in preeclampsia. sFlt-1 acts as a potent vascular endothelial growth factor (VEGF) and placental growth factor (PlGF) antagonist by binding to these molecules and thereby reducing the free circulating levels of VEGF and PlGF. Decreased levels of circulating VEGF and PlGF appear to result in reversal of physiological vasodilation, leading to hypertension [8]. Several studies have reported that the sFlt-1/PlGF ratio can be used to identify patients at risk of preeclampsia [9,10]. Verlohren *et al.*[9] reported that a sFlt-1/PlGF ratio of ≥ 85 (Elecsys; Roche Diagnostics GmbH, Mannheim, Germany) predicted preeclampsia. However, a lower cutoff value could yield a more sensitive diagnostic test.

In high-risk women, persistently increased uterine artery (UtA) resistance during the first half of pregnancy is associated with a high risk of early-onset preeclampsia [7]. A combination of UtA Doppler ultrasonography measurements (pulsatility index and mean arterial pressure) and biochemical markers at 11 to 13 weeks of gestation effectively identified women at high risk of hypertensive disorders in pregnancy [11]. Yu *et al.*[12] reported that in low-risk pregnancies, preeclampsia associated with delivery at or beyond 34 weeks of gestation was most effectively predicted by a combination of ultrasonography findings and maternal factors. However, the ability of various biophysical markers to predict preeclampsia in low-risk pregnancies should be examined more thoroughly.

The integrated test for fetal Down’s syndrome uses a combination of placental biomarkers. Among these markers, the levels of pregnancy-associated plasma protein (PAPP-A) and inhibin-A are positively associated with subsequent preeclampsia [13-16]. However, the screening efficacy of these markers for preeclampsia remains to be determined. This study evaluated the ability of the PAPP-A and inhibin-A levels, sFlt-1/PIGF ratio, and UtA Doppler ultrasonography measurements to predict preeclampsia in low-risk pregnancies, and established a cutoff value for the sFlt-1/PlGF ratio determined using the Elecsys platform.

## Methods

### Study population

This prospective study analyzed the risk of preeclampsia according to the results of blood sample analyses in pregnant women at low risk of preeclampsia. The study included women who received regular antenatal care at the prenatal care unit of Cha Hospital in Seoul, Korea between April 2011 and December 2011. All patients were recruited before 10 weeks of gestation, based on measurement of the fetal crown-rump length. All subjects underwent the integrated test for fetal Down’s syndrome, UtA Doppler ultrasonography at 20 to 24 weeks of gestation, and calculation of the sFlt-1/PlGF ratio at 24 to 27 and 34 to 37 weeks of gestation. Women were excluded if they were aged ≥ 40 years or had a multifetal pregnancy, chronic hypertension, prior history of preeclampsia, pregestational diabetes mellitus, gestational diabetes mellitus, delivery before 35 weeks of gestation, early-onset preeclampsia (before 35 weeks of gestation), or body mass index (BMI) ≥ 25 kg/m^2^. All the cases of preeclampsia included in the study were diagnosed with severe preeclampsia after 35 weeks of gestation (late-onset preeclampsia), and the time of diagnosis was almost the same as the time of delivery.

Preeclampsia was defined according to the National High Blood Pressure Education Program Working Group on High Blood Pressure in Pregnancy criteria. Hypertension was defined as repeated systolic blood pressure measurements of ≥ 140 mmHg (Korotkoff phase 1) and diastolic blood pressure measurements of ≥ 90 mmHg (Korotkoff phase 5). Proteinuria was defined as ≥ 300 mg of protein in a 24-hour urine collection sample, or repeated ≥ 1+ proteinuria on dipstick urinalysis [17]. Preeclampsia was defined as severe if (1) the systolic blood pressure was > 160 mmHg or the diastolic blood pressure was > 110 mmHg on at least two occasions after 20 weeks of gestation, or (2) there was ≥ 5 g of protein in a 24-hour urine collection specimen or ≥ 3+ proteinuria on dipstick urinalysis of two samples collected at least 4 hours apart. The time of onset of preeclampsia was defined as the date of diagnosis. Normal pregnancy was defined as term delivery with no documented concerns regarding hypertension or proteinuria before or after delivery.

### Ethics statement

All women provided written informed consent before the collection of blood samples. The collection and use of samples for the purposes of this study were approved by the Institutional Review Board of CHA Gangnam Medical Center, CHA University.

### Laboratory methods

All women underwent the integrated test for fetal Down’s syndrome. Maternal serum samples were obtained at 11^+0^ to 13^+6^ and 15^+0^ to 20^+6^ weeks of gestation for measurement of the plasma PAPP-A level and the serum levels of the quadruple test markers: alpha-fetoprotein (MSAFP), unconjugated estriol, human chorionic gonadotropin (hCG), and inhibin-A. Markers were measured using a UniCel DxI 800 analyzer (Beckman Coulter Inc., Fullerton, CA, USA) and the values were transformed to multiples of the median (MoM) after adjusting for gestational age and maternal BMI.

To measure the plasma levels of sFlt-1 and PlGF, venous blood was collected in silicon-coated glass tubes at 24^+0^ to 27^+6^ and 34^+0^ to 37^+6^ weeks of gestation. After clotting, the samples were centrifuged and plasma was stored at -80°C. The sFlt-1 and PlGF levels of each sample were measured simultaneously using the fully automated Roche Diagnostics Elecsys assay (Roche Diagnostics, Penzberg, Germany), and the sFlt-1/PlGF ratio was calculated.

### Uterine artery Doppler ultrasonography

UtA Doppler transabdominal ultrasonography with color flow mapping was performed at 20 to 24 weeks of gestation. The bilateral UtA Doppler impedance indices were recorded according to the Fetal Medicine Foundation guidelines [12], including the mean pulsatility index, resistance index, systolic/diastolic ratio, and notching.

### Statistical analysis

Data are expressed as the median (range) and were analyzed using non-parametric methods.

Weighted log10-linear regression was used to calculate the median values for the levels of sFlt-1, PlGF, PAPP-A, alpha-fetoprotein (MSAFP), β-hCG, unconjugated estriol, and inhibin-A, and the highest uterine artery pulsatility index, mean uterine artery pulsatility index, highest uterine artery resistance index, mean uterine artery resistance index, and nuchal translucency thickness, as a function of fetal crown-rump length and maternal weight for the 254 subjects without preeclampsia. The results for all subjects were then expressed as MoM values. Repeated analysis of variables was used to calculate the effects of gestational age on variables. Receiver operating characteristic (ROC) curve analysis was used to determine the detection rate (DR) of each marker for subsequent late-onset preeclampsia at a fixed false-positive rate (FPR). Logistic regression analysis was used to calculate the *a posteriori* risk of preeclampsia for each patient using the panel of available markers expressed in terms of MoM and parity.

The logistic regression equation used was ln(y) = α + β_1_χ_1_ + β_2_χ_2_ + … β_n_χ_n_ where α is the constant of the model (the odds of preeclampsia without risk factors or when the risk factors assume the lowest risk value), β is the coefficient associated with the risk factor, χ is the risk factor, and y is the odds of preeclampsia. Therefore, odds = exp(y) and risk = odds/(1 + odds).

Analyses were performed using the Statistical Package for the Social Sciences version 19.0 (SPSS Inc., Chicago, IL, USA) and Power Analysis and Sample Size version 11 (NCSS LLC, Kaysville, UT, USA).

## Results

**Figure 1:**
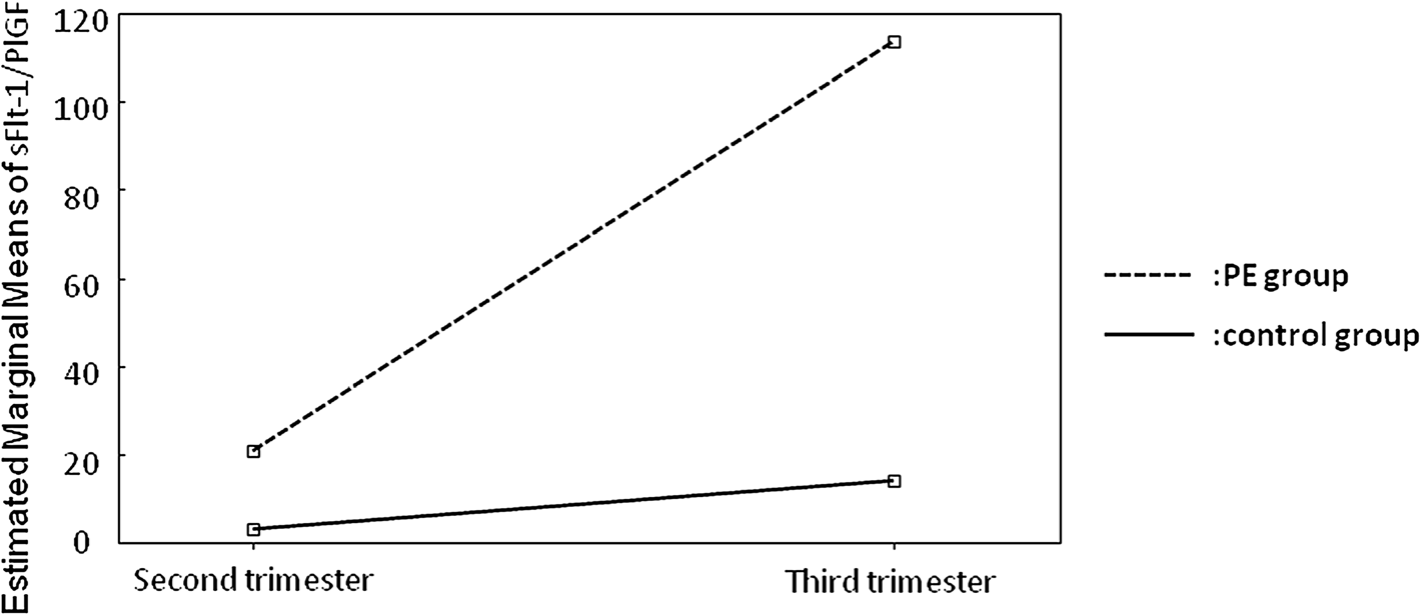
**Slope of the sFlt-1/PlGF ratio.** Results of repeated measures analysis of variance comparing the second trimester sFlt-1/PlGF ratio (sFlt-1/PlGF^1^) with the third trimester sFlt-1/PlGF ratio (sFlt-1/PlGF^2^). Dotted line: patients with preeclampsia, solid line: patients without preeclampsia.

A total of 262 women were enrolled in this study, of which 8 developed late-onset preeclampsia. The characteristics of subjects with and without preeclampsia and the distributions of the studied markers are shown in Table 1. There were significant differences in maternal weight before pregnancy and at delivery, BMI, infant birth weight, and parity between patients with and without preeclampsia. The BMI before pregnancy was significantly higher (p = 0.010), infant birth weight was significantly lower (p < 0.001), and delivery time was significantly earlier (p < 0.001) in patients with preeclampsia than in patients without preeclampsia. Small-for-gestational-age infants (birth weight below the 10^th^ percentile) were significantly more frequent in patients with preeclampsia than in patients without preeclampsia (p = 0.006). The MoM values of all the biochemical markers except for the MSAFP level, and of nuchal translucency thickness, were significantly different between patients with and without preeclampsia (Table 2). UtA Doppler ultrasonography measurements were not significantly different between patients with and without preeclampsia. Figure 1 shows the results of repeated measures analysis of variance comparing the second and third trimester sFlt-1/PlGF ratios in patients with and without preeclampsia. The ratios were significantly associated with time (within-subjects effect) and the development of preeclampsia (between-subjects effect) (both p < 0.001, sphericity test).

**Table 1:** Characteristics of the subjects

**Variables**	**Preeclampsia (n = 8)**	**Unaffected group (n = 254)**	*P*‒value *
Maternal age, yrs	33 (25–37)	33 (20–39)	0.872
Nulliparous, %	66.1	50	0.779
Prepregnancy weight (kg)	62 (50–71)	53 (40–75)	0.018
BMI, kg/m^2^, mean	22.9 (19.05–24.86)	20.2 (15.24–24.9)	0.010
Weight at delivery	78 (61.7–107)	65 (43.5–102.2)	0.007
Infant birth weight,gm	2385 (1760–3100)	3160 (2100–4000)	<0.001
Small for gestational age(%)	37.5	8.7	0.006
Gestational age at delivery	35.5 (35–40)	38.5 (35–41)	<0.001

Data are expressed as median (minimum–maximum) or percentage.

*Mann–Whitney *U* Test or Fisher’s test.

**Table 2:** Distributions of the variables of interest

**variable**	**Preeclampsia (n = 8)**	**Unaffected group (n = 254)**	**P value**
Median gestational age (weeks)	11 (10–13)	11 (10–13)	0.651
PAPP-A, MoM	0.63 (0.34–1.67)	1.00 (0.20–3.85)	0.046
NT, MoM	1.05 (0.81–1.28)	1.00 (0.45–1.99)	0.724
Median gestational age	16 (15–17)	16 (14–18)	0.784
MSAFP, MoM	1.00 (0.41–4.38)	1.10 (0.55–2,96)	0.550
hCG,MoM	1.66 (1.16–2.68)	1.00 (0.20–6.53)	0.001
uE3, MoM	0.97 (0.69–1.28)	1.00 (0.46–1.83)	0.521
Inhibin A, MoM	1.86(0.82–2.96)	1.00 (0.39–3.676)	0.007
Median gestational age	26 (24–27)	27 (25–27)	0.378
sFlt-1^1^, (pg/ml)	2360 (932–4435)	1296 (166–5710)	0.001
PlGF^1^, (pg/ml)	219 (34.25–698)	491 (91.37–2243)	0.016
sFlt-1/PlGF^1^	11.9 (1.7–70.9)	2.6 (0.3–12)	0.001
Median gestational age	34 (34–36)	36 (34–37)	<0.001
sFlt-1^2^, (pg/ml)	6275 (4307–12835)	2334 (71.61–50475)	<0.001
PlGF^2^, (pg/ml)	115.9 (16.48–284.9)	287.95 (6.60–1749)	0.001
sFlt-1/PlGF^2^	76.3 (15.1–352.7)	8 (0.8–156)	<0.001
Uterine artery doppler			
Mean RI	0.55 (0.48–0.77)	0.57 (0.37–0.83)	0.972
Mean PI	0.94 (0.71–1.86)	0.97 (0.52–2.00)	0.949
Mean SD	2.25 (1.95–4.25)	2.35 (1.47–5.91)	0.913
Highest RI	0.58 (0.53–0.77)	0.62 (0.38–0.88)	0.526
Highest PI	1.03 (0.79–1.86)	1.06 (0.54–2.76)	0.808
Highest SD	2.40 (2.10–4.30)	2.58 (1.63–8.58)	0.642
Notch (%)	0.4	12.5	0.072

Data are expressed as median (minimum–maximum).

PAPP-A: pregnancy-associated plasma protein-A, NT: nuchal translucency thickness, MSAFP: alpha-fetoprotein, hCG: human chorionic gonadotropin, uE3: unconjugated estriol, sFlt-1^1^: second trimester sFlt-1, PlGF^1^: second trimester PlGF, sFlt-1/PlGF^1^: second trimester sFlt-1/PlGF ratio, sFlt-1^2^: third trimester sFlt-1, PlGF^2^: third trimester PlGF, sFlt-1/PlGF^2^: thirst trimester sFlt-1/PlGF ratio.

**Figure 2:**
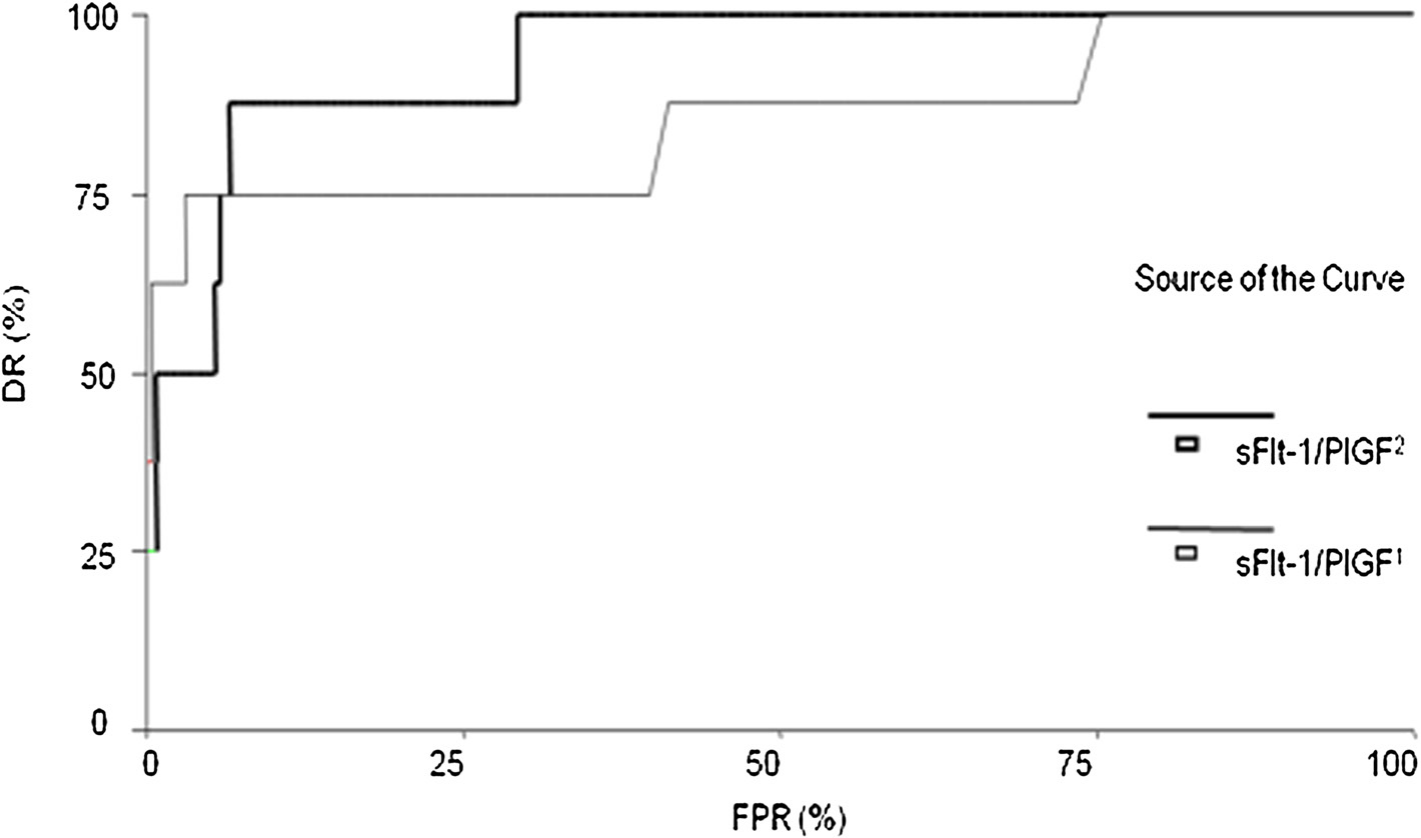
**Univariate ROC curves for the second and third trimester sFlt-1/PlGF ratios.** The second trimester sFlt-1/PlGF ratio (sFlt-1/PlGF^1^) and third trimester sFlt-1/PlGF ratio (sFlt-1/PlGF^2^) were the best markers for predicting preeclampsia.

The univariate ROC curve analyses for each of the variables evaluated are shown in Table 3. The third trimester sFlt-1/PlGF ratio yielded the highest DR of 87.5% at a fixed FPR of 10% (p < 0.001), followed by the second trimester sFlt-1/PlGF ratio with a DR of 75% (p = 0.001). Of the single markers, the sFlt-1 level yielded the best DR (75%) at a fixed FPR of 10%, followed by the PlGF level (62.5%) and the PAPP-A level (50%). Even though the number of preeclampsia cases was small, the two-tailed power of the data with a Type I error rate of 5% ranged from 86% to 99% for all the ROC curves. For logistic regression analysis, an odds ratio of > 2.5 was required to achieve sufficient power with a type I error rate of 5% at the given incidence of preeclampsia (8/262, 3%). Figure 2 shows the univariate ROC curves for the second and third trimester sFlt-1/PlGF ratios, which had the best performance for predicting preeclampsia out of the available markers.

**Table 3:** Univariate ROC curve analysis for each marker and the detection rate (DR) for preeclampsia at a fixed false-positive rate (FPR) of 10% and 5%

**Variable**	**Cutoff**^**1**^	**DR**^**1**^	**Cutoff**^**2**^	**DR**^**2**^	**Area**	**SE**	**p-value**	**95% CI**	
BMI, kg/m^2^	23.6	37.5	24.3	25	0.769	0.086	0.010	0.600	0.937
PAPP-A, MoM	0.58	50	0.49	50	0.707	0.116	0.046	0.480	0.935
Inhibin A, MoM	1.76	50	2.18	50	0.780	0.095	0.007	0.593	0.967
sFlt-1^1^, (pg/ml)	2358	75	3064	25	0.842	0.080	0.001	0.686	0.998
sFlt-1^2^	4680	75	6040	50	0.944	0.020	<0.001	0.906	0.983
PlGF^1^, (pg/ml)	257	62.5	200	37.5	0.751	0.103	0.016	0.549	0.953
PlGF^2^	113.4	50	86.5	37.5	0.847	0.060	0.001	0.729	0.965
sFlt-1/PlGF^1^	6.05	75	7.0	75	0.851	0.092	0.001	0.670	1.000
sFlt-1/PlGF^2^	28.2	87.5	43.7	50	0.939	0.033	<0.001	0.873	1.000

Cutoff^1^: cutoff value at a fixed FPR of 10%, DR^1^: DR at a fixed FPR of 10%, Cutoff^2^: cutoff value at a fixed FPR of 5%, DR^2^: DR at a fixed FPR of 5%, PAPP-A: pregnancy-associated plasma protein-A, sFlt-1^1^: second trimester sFlt-1, PlGF^1^: second trimester PlGF, sFlt-1/PlGF^1^: second trimester sFlt-1/PlGF ratio, sFlt-1^2^: third trimester sFlt-1, PlGF^2^: third trimester PlGF, sFlt-1/PlGF^2^: third trimester sFlt-1/PlGF ratio, SE: standard error.

**Figure 3:**
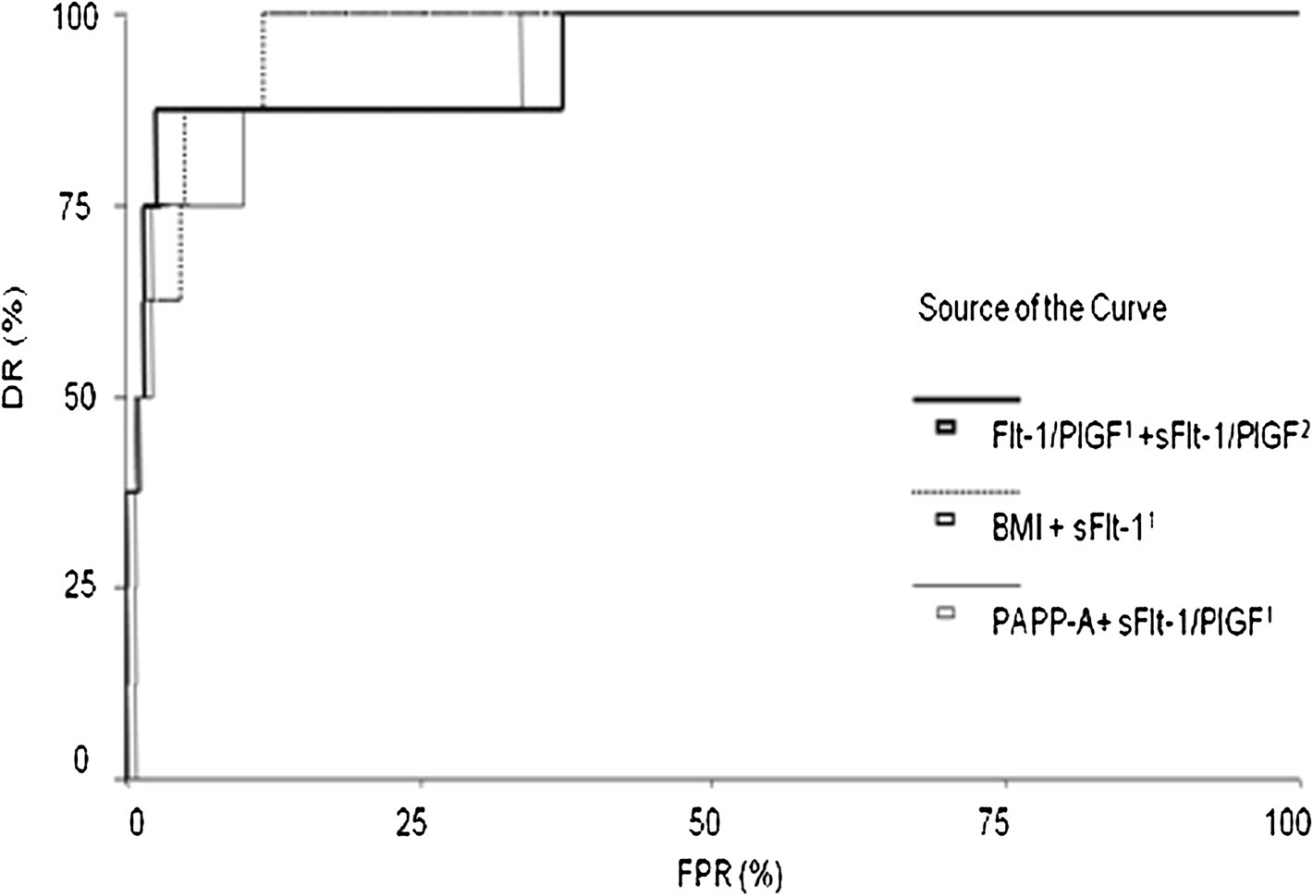
**Multivariate ROC curves for combination models.** PAPP-A + sFlt-1/PlGF^1^, BMI + sFlt-1^1^, and sFlt-1/PlGF^1^ + sFlt-1/PlGF^2^ were the best combinations for predicting preeclampsia. PAPP-A: pregnancy-associated plasma protein-A, sFlt-1^1^: second trimester sFlt-1, sFlt-1/PlGF^1^: second trimester sFlt-1/PlGF ratio, sFlt-1/PlGF^2^: third trimester sFlt-1/PlGF ratio.

Multivariate ROC curves were constructed using the logistic regression analysis results (Table 4). The best performances were achieved by the combination of the PAPP-A level and the second trimester sFlt-1/PlGF ratio, and the combination of the second and third trimester sFlt-1/PlGF ratios. It is worth noting that the combination of the PAPP-A level and the second trimester sFlt-1/PlGF ratio had a DR of 87.5% (at a fixed FPR of 10%), and the second trimester sFlt-1/PlGF ratio alone had a DR of 75% (at a fixed FPR of 10%). The addition of BMI to the second trimester sFlt-1 level increased the DR from 75% to 87.5%. The DR of the combination of the second and third trimester sFlt-1/PlGF ratios was not improved by the addition of any other marker. Interestingly, the cutoff for high risk of preeclampsia was similar for each of the five models, ranging from 2.2% to 5%. Figure 3 shows multivariate ROC curves for combination models. Pattern analysis results for the cases detected by the logistic models are shown in Table 5 (right columns) and Table 6. Three cases were detected by three of the models: the combination of the PAPP-A level and second trimester sFlt-1/PlGF ratio, the combination of the BMI and the second trimester sFlt-1 level, and the combination of the second and third trimester sFlt-1/PlGF ratios. Two cases were detected by all five models. The combination of the PAPP-A and inhibin-A levels failed to detect two of the remaining three cases. However, the three remaining cases were each detected by at least two models (Table 6). Parity (nulliparous versus parous) did not add any discriminatory power and was therefore excluded from the logistic models. The risk estimates for each of the eight cases of preeclampsia according to the logistic models are shown in Table 5. The different models provided quite different risk estimates for preeclampsia for the same patient. For example, Cases 4 and 218 had a risk estimation range of more than 90% (from 0.59% to 99.32% and from 5.46% to 99.99%, respectively). This indicates that more cases need to be evaluated in future prospective studies to identify the best cutoff values for detecting patients at high risk of preeclampsia.

**Table 4:** ROC curve analyses for combinations of markers and the DR for preeclampsia at a fixed FPR of 10% and 5% using a logistic regression model

**Variable**	**Cutoff**^**1**^	**DR**^**1**^	**Cutoff**^**2**^	**DR**^**2**^	**Area**	**S E**	**p-value**	**95% CI**	**Variable**
PAPP-A + Inhibin A	5.01%	50	9.19%	37.5	0.812	0.093	0.003	0.629	0.995
PAPP-A + sFlt-1/PlGF^1^	2.85%	87.5	4.72%	87.5	0.969	0.015	<0.001	0.940	0.999
PAPP-A + sFlt-1/PlGF^2^	2.62%	62.5	4.07%	50	0.917	0.036	<0.001	0.847	0.988
BMI + sFlt-1^1^	4.6%	87.5	8.9%	75	0.937	0.039	<0.001	0.861	1.000
sFlt-1/PlGF^1^ + sFlt-1/PlGF^2^	2.2%	87.5	3.4%	87.5	0,946	0.043	<0.001	0.862	1.000

The exploratory variable in each case is the risk determined by the logistic equation.

Cutoff^1^: cutoff value at a fixed FPR of 10%, DR^1^: DR at a fixed FPR of 10%, Cutoff^2^: cutoff value at a fixed FPR of 5%, DR^2^: DR at a fixed FPR of 5%, PAPP-A: pregnancy-associated plasma protein-A, sFlt-1^1^: second trimester sFlt-1, sFlt-1/PlGF^1^: second trimester sFlt-1/PlGF ratio, sFlt-1/PlGF^2^: third trimester sFlt-1/PlGF ratio, SE: standard error.

**Table 5:** Estimated risk (%) for each case of preeclampsia for each model at a fixed FPR of 10%

**Cases ID**	**PAPP-A + Inhibin A**	**PAPP-A + sFlt-1/PlGF**^**1**^	**PAPP-A + sFlt-1/PlGF**^**2**^	**BMI + sFlt-1**^**1**^	**sFlt-1/PlGF**^**1**^**+ sFlt-1/PlGF**^**2**^	Pattern*				
38	4.43	2.59	55.44	0.79	7.25	0	0	1	0	1
51	18.64	4.76	2.62	5.30	0.64	1	1	1	1	0
147	2.86	5.40	1.62	51.4	19.85	0	1	0	1	1
137	36.53	33.60	38.30	19.1	31.95	1	1	1	1	1
144	2.96	36.02	3.11	42.9	20.90	0	1	1	1	1
4	0.59	99.32	1.88	40.3	97.83	0	1	0	1	1
218	5.46	99.99	80.35	20.8	99.99	1	1	1	1	1
153	26.96	100	99.92	30.6	100	1	1	1	1	1

*0 = missed case, 1 = detected case.

PAPP-A: pregnancy-associated plasma protein-A, sFlt-1^1^: second trimester sFlt-1, PlGF^1^: second trimester PlGF, sFlt-1/PlGF^1^: second trimester sFlt-1/PlGF ratio, sFlt-1^2^: third trimester sFlt-1, PlGF^2^: third trimester PlGF, sFlt-1/PlGF^2^: third trimester sFlt-1/PlGF ratio.

**Table 6:** Patterns analysis for detection of preeclampsia for each model at a fixed FPR of 10%

**Number of cases**	Detection model of preeclampsia*				
	**PAPP-A + Inhibin A**	**PAPP-A + sFlt-1/PlGF**^**1**^	**PAPP-A + sFlt-1/PlGF**^**2**^	**BMI + sFlt-1**^**1**^	**sFlt-1/PlGF**^**1 **^**+ sFlt-1/PlGF**^**2**^
1	0	0	1	0	1
2	0	1	0	1	1
1	0	1	1	1	1
1	1	1	1	1	0
3	1	1	1	1	1

*0 = missed case, 1 = detected case.

PAPP-A: pregnancy-associated plasma protein-A, sFlt-1^1^: second trimester sFlt-1, PlGF^1^: second trimester PlGF, sFlt-1/PlGF^1^: second trimester sFlt-1/PlGF ratio, sFlt-1^2^: third trimester sFlt-1, PlGF^2^: third trimester PlGF, sFlt-1/PlGF^2^: third trimester sFlt-1/PlGF ratio.

## Discussion

This study used combinations of maternal and biochemical markers to identify women at high risk of preeclampsia in a low-risk population. We focused on late-onset preeclampsia in a low-risk population because the prevalence of late-onset preeclampsia is much higher than the prevalence of early-onset preeclampsia [18]. Early-onset preeclampsia is usually associated with placental dysfunction, reduction in placental volume, intrauterine growth restriction, abnormal uterine and umbilical artery Doppler ultrasonography findings, low birth weight, multi-organ dysfunction, perinatal death, and adverse maternal and neonatal outcomes [19,20]. Late-onset preeclampsia is more often associated with a normal placenta, larger placental volume, normal fetal growth, normal uterine and umbilical artery Doppler ultrasonography findings, normal birth weight, and more favorable maternal and neonatal outcomes [19,21,22]. Considering the heterogeneous pathophysiology, no single screening test is sufficient for predicting preeclampsia.

Our results indicate that combination of the Elecsys sFlt-1/PlGF ratio with the pre-pregnancy BMI and biochemical markers during pregnancy may improve the sensitivity of predicting preeclampsia in a low-risk population. The DRs for individual markers ranged from 37.5% to 87.5% for a fixed FPR of 10%, and from 25% to 75% for a fixed FPR of 5%. After combining markers, the estimated DR ranged from 50% to 87.5% for a fixed FPR of 10%, and from 37.5% to 87.5% for a fixed FPR of 5%. Combining markers had a moderate benefit at a fixed FPR of 5%. The second and third trimester sFlt-1 levels had the highest DRs among the single markers, but the sFlt-1/PlGF ratio was a stronger predictor of preeclampsia than either marker alone. The third trimester sFlt-1/PlGF ratio had the highest DR of the individual variables evaluated, but was not sufficient as a single screening method for early detection of preeclampsia.

ROC curve analysis showed that BMI was the strongest clinical predictor of preeclampsia with a DR of 37.5% at a fixed FPR of 10% and a DR of 25% at a fixed FPR of 5%. A previous study reported that increased early pregnancy insulin resistance was independently associated with subsequent preeclampsia [23]. We found that BMI predicted preeclampsia (p = 0.010) at a cutoff value of 23.64 kg/m^2^ at a fixed FPR of 10% and 24.26 kg/m^2^ at a fixed FPR of 5%, even in a low-risk population with BMI < 25 kg/m^2^. This result is consistent with previous reports that obese women are at increased risk of developing preeclampsia [24,25].

Duckitt and Harrington [26] performed a systematic review of 52 studies and found that nulliparity was a risk factor for preeclampsia (relative risk: 2.91). A prospective cohort study also found that nulliparous women had higher blood pressure levels throughout pregnancy and higher risks of uterine artery notching and gestational hypertensive disorders than other women [27]. However, no association between preeclampsia and parity was observed in this study (p = 0.779). This difference may result from differences in ethnicity and marital status between the subjects in the different studies, or the relatively small number of cases in this study.

The integrated test for fetal Down’s syndrome is based on the levels of several first and second trimester fetoplacental markers. PAPP-A is a large, highly glycosylated protein that is produced by developing trophoblast cells. PAPP-A has been shown to be a syncytiotrophoblast-derived insulin-like growth factor binding protein protease [28]. A multicenter study of 8,839 women demonstrated a significant relationship between a PAPP-A level at or below the 5^th^ percentile and intrauterine growth restriction, preterm delivery, preeclampsia, and stillbirth [13]. Inhibin-A is a glycoprotein hormone that is a member of the transforming growth factor-β family. The placenta is the primary source of this circulating protein during pregnancy, and its concentration increases in the third trimester of uncomplicated pregnancies [29]. Although an increased serum inhibin-A level is significantly associated with subsequent preeclampsia, the inhibin-A level has poor sensitivity for predicting preeclampsia [14,15]. Nevertheless, we found that the inhibin-A level was a more sensitive marker for predicting subsequent preeclampsia than the hCG level in the second trimester. Addition of the hCG level to the inhibin-A level did not improve the screening efficacy for preeclampsia in a previous study [16]. Significantly lower PAPP-A levels and higher inhibin-A and hCG levels were observed in women who developed preeclampsia than in those who did not, but the levels of unconjugated estriol and MSAFP had no predictive power. At a fixed FPR of 10% and 5%, the PAPP-A and inhibin-A levels both had DRs of 50%. In our hospitals, the integrated test for fetal Down’s syndrome is routinely offered to all women. This test includes the PAPP-A level at 11 to 13 weeks of gestation and the inhibin-A level at 15–20 weeks of gestation. These biochemical markers for Down’s syndrome could potentially be used to improve the prediction of preeclampsia.

Recent studies have shown that UtA Doppler waveform analysis can identify women at risk of adverse pregnancy outcomes in the second trimester [30-32]. As described above, abnormal placentation is considered to play a central role in the pathogenesis of preeclampsia. Shallow cytotrophoblast interstitial invasion and failure of endovascular invasion result in increased vascular resistance and decreased placental perfusion [32]. However, this mechanism is more relevant in early-onset preeclampsia than in late-onset preeclampsia. A recent study proposed that 22 to 25 weeks of gestation is the best time for predicting preeclampsia by uterine artery Doppler velocimetry data [33]. Costa *et al.*[34] reported that second trimester UtA Doppler ultrasonography measurements had high sensitivity for predicting preeclampsia in a low-risk population, but only a 29% positive predictive value. A low positive predictive value for late-onset disease can complicate screening in a low-risk population. In our study, we found no significant association between UtA Doppler ultrasonography measurements and preeclampsia. Our small sample size and the fact that all cases of preeclampsia were late-onset disease might have contributed to our failure to detect a significant association between UtA Doppler ultrasonography measurements and preeclampsia.

The Elecsys system is an immunoassay system that measures plasma sFlt-1 and PlGF levels. Although it was developed for diagnostic testing of patients with preeclampsia, a carefully established cutoff value could potentially enable detection of subclinical preeclampsia. A recent study demonstrated patients with preeclampsia or the syndrome of hemolysis, elevated liver enzymes, and low platelet count with a high sFlt-1/PlGF ratio were at significantly increased risk of imminent delivery, and the authors proposed that the sFlt-1/PlGF ratio be used as a prognostic marker [10]. In our study, blood samples for the Elecsys assay were collected at 24^+0^ to 27^+6^ and 34^+0^ to 37^+6^ weeks of gestation. The sFlt-1 level yielded a DR of 25% at a fixed FPR of 10%, and a DR of 75% at a fixed FPR of 5%. The PlGF level yielded a lower DR than the sFlt-1 level at a fixed FPR of 5%, in the first trimester but not in the second trimester. The second trimester sFlt-1/PlGF ratio yielded a DR of 75% at a fixed FPR of 10% (cutoff value: 6.05) and 5% (cutoff value: 7.0). The third trimester sFlt-1/PlGF ratio yielded a DR of 87.5% at a fixed FPR of 10% (cutoff value: 28.15) and a DR of 50% at a fixed FPR of 5% (cutoff value: 43.70). The Elecsys instructions recommend using an sFlt-1/PlGF ratio of > 85 (independent of gestational age) for diagnosing preeclampsia. The results of this study show that the sFlt-1/PlGF ratio is a useful predictor of late-onset preeclampsia. Ohkuchi *et al.*[35] reported that a cutoff value of 45 for the Elecsys sFlt-1/PlGF ratio gave 100% sensitivity and 95% specificity for detecting preeclampsia in a Japanese population. Soto *et al.*[36] reported that women with late-onset preeclampsia had significantly lower PlGF and soluble VEGF receptor-2 levels, lower PlGF/soluble endoglin and PlGF/soluble VEGF receptor-1 ratios, and higher soluble VEGF receptor-1 and soluble endoglin levels than normal pregnant women. Evidence of an imbalance between angiogenic and anti-angiogenic factors in preeclampsia has consistently been observed in women of Hispanic, Caucasian, Asian, African, and African-American origin, and therefore appears to be a consistent finding of this disorder [36].

In this study, small-for-gestational-age infants were usually associated with preeclampsia. In patients without preeclampsia, the median sFlt-1/PlGF ratio at 34 to 37 weeks of gestation was low but had a very wide range (median: 8; range: 0.8–156). Four patients had an sFlt-1/PlGF ratio of > 85. Among these patients, the mean UtA pulsatility index was above the 95^th^ percentile for gestational age [37] in only one case with a small-for-gestational-age infant.

In this study, the combination of the second and third trimester sFlt-1/PlGF ratios and the combination of the PAPP-A level and the second trimester sFlt-1/PlGF ratio yielded the best DRs. The sFlt-1/PlGF ratio measured shortly before delivery at or beyond 34 weeks of gestation has little value as a screening test, because delivery is an effective treatment for late-onset preeclampsia. However, if the second trimester sFlt-1/PlGF ratio is equivocal and the patient does not have preeclampsia at 34 weeks of gestation, serial measurements may help clinicians to detect and manage emerging late-onset preeclampsia. The combination of the PAPP-A level and the second trimester sFlt-1/PlGF ratio is therefore the best marker for early detection of late-onset preeclampsia.

Akolekar *et al.*[38] developed an effective first-trimester screening model for preeclampsia using the UtA pulsatility index, mean arterial pressure, and PAPP-A and PlGF MoM values. Their algorithms detected 95.3% of cases of early-onset preeclampsia and 45.6% of cases of late-onset preeclampsia at a fixed FPR of 10.9%. Saxena *et al.*[39] reported that the mean third trimester sFlt-1 level was significantly higher (p = 0.002), and the mean first trimester sFlt-1 level was significantly lower (p = 0.03), in women who developed preeclampsia than in women with normal pregnancies. The serial measurements of the sFlt-1/PlGF ratio used in this study enabled a much higher rate of detection of late-onset preeclampsia than the 45.6% described above.

A weak point of our study is the different risk estimation results obtained when different combinations of markers were used in the same subject. This is probably due to the low number of patients with preeclampsia, and the fact that preeclampsia results from various pathophysiologic mechanisms that may alter the markers in different ways. However, three of the eight cases were detected by all the models used, and all the cases were detected by at least two of the models. The strong points of the study include its prospective design and the evaluation of several markers for predicting preeclampsia. The combinations used in this study appear to have higher predictive accuracy than the combination of first-trimester maternal risk factors and mean arterial pressure used in the large prospective study by Poon *et al.*[40], which reported a 52% detection rate for preeclampsia at a 10% false-positive rate. A larger number of patients may have resulted in different accuracy rates for the combinations evaluated in our study. However, there was a steep increase in the sFlt-1/PlGF ratio in patients with preeclampsia (Figure 1), which is consistent with previously reported results [41], and we expect that the main conclusions would not change with a larger study population. Although the slope of the sFlt-1/PlGF ratio was not used in our algorithms, this value may be useful for enhancing predictive accuracy in a future larger scale study.

## Conclusions

The Elecsys sFlt-1/PlGF ratio allows identification of low-risk pregnancies at high risk of developing preeclampsia. A cutoff value of 6.05 in the second trimester and 28.15 in the third trimester had the best ability to predict preeclampsia at a fixed FPR of 10%. The PAPP-A level and BMI can be used in combination with the sFlt-1/PlGF ratio to increase the rate of detection of preeclampsia. Further studies are required to assess the usefulness of our combined screening test in low-risk populations.

## Competing interests

All authors declare that they have no competing interests.

## Authors’ contributions

DHC and KJL conceived of the study. HJP, DHC, KJL and SHK participated in the study design. AF and MZ performed the statistical analyses. HJP, AF and MZ interpreted the data. HJP drafted and revised the manuscript. HJP, DHC, KJL, SHK, SSS, JYK and YKC made substantial contributions to the acquisition of data. DHC, KJL, YWJ and AF critically reviewed the manuscript. All authors read and approved the final manuscript.

## Pre-publication history

The pre-publication history for this paper can be accessed here:

http://www.biomedcentral.com/1471-2393/14/35/prepub
